# The *LncRNA401*-*LrWRKY70* Module Regulates the Blue-Purple Flower Color Formation in *Lycoris*

**DOI:** 10.3390/plants15081223

**Published:** 2026-04-16

**Authors:** Cai Qin, Pengchong Zhang, Qing Yang, Yuhong Zheng, Meng Qi, Tianyi Wang, Qiujie Wang, Yi Wang, Chongde Sun, Xiao Shen, Ting Lu, Dong Meng, Haizhen Zhang

**Affiliations:** 1 College of Agriculture and Biotechnology, Zhejiang University, Hangzhou 310058, China; qincai0324@163.com (C.Q.); adesun2006@zju.edu.cn (C.S.); 2 Hangzhou Botanical Garden, Hangzhou West Lake Academy of Landscape Science, No. 1 Tao Yuanling, Xihu District, Hangzhou 310013, China; zhang-pengchong@163.com (P.Z.); shenxiao32167@163.com (X.S.); hzlj2016@hzbg.cn (T.L.); 3 College of Forestry, Beijing Forestry University, Beijing 100083, China; yang.qing1020@163.com (Q.Y.); qimqmm@163.com (M.Q.); wangty02@163.com (T.W.); wqj969842264@163.com (Q.W.); 4 Jiangsu Key Laboratory for Conservation and Utilization of Plant Resources, Institute of Botany, Jiangsu Province and Chinese Academy of Sciences, China No. 1 Qianhuhoucun, Xuanwu District, Nanjing 210014, China; zhengyuhong@cnbg.net; 5 International Center for Bamboo and Rattan Sanya Research Base, Sanya 572000, China; wangyisy@icbr.ac.cn

**Keywords:** *Lycoris*, blue-purple, kaempferol, LrWRKY70, *LncRNA401*

## Abstract

*Lycoris* plants are known for their diverse flower colors, but the molecular mechanisms behind these variations remain unclear. In this study, we first used the CIELAB system to precisely measure flower color. We objectively defined the petals of *Lycoris sprengeri* as blue-purple (Bp) and compared them with the white petals of *Lycoris longituba* (W) and the red petals of *Lycoris radiata* var. *pumila* (R). Metabolomic analysis showed that specific kaempferol glycosides, including kaempferol-3-*O*-sophoroside and lonicerin, accumulated significantly in the blue-purple petals. Transcriptomic analysis revealed that genes related to flavonoid biosynthesis were generally more active in the colored petals (Bp and R). However, different expression patterns of key hydroxylase genes created a metabolic split. Specifically, the blue-purple petals showed high expression of *LrF3′5′H* (directing synthesis toward delphinidin) and *LrFLS* (promoting kaempferol accumulation), whereas the red petals mainly expressed *LrF3′H* (leading to cyanidin synthesis). Further investigation identified LrWRKY70 as a core transcription factor highly correlated with these flavonoid pathway genes. Crucially, we discovered a new long non-coding RNA, *LncRNA401*, located downstream of the *LrWRKY70* antisense strand. It showed a strong positive correlation with *LrWRKY70*. Functional verification through transient overexpression demonstrated that *LncRNA401* significantly increased the expression of *LrWRKY70*. This, in turn, broadly activated downstream flavonoid biosynthesis genes, including *LrCHS*, *LrF3′5′H*, *LrFLS*, and *LrDFR*. This cascade ultimately promoted the synthesis of anthocyanins and kaempferol derivatives, resulting in the unique blue-purple phenotype. Our results reveal a novel *LncRNA401*-*LrWRKY70* regulatory module. This module plays a key role in metabolic reprogramming for flower color formation in *Lycoris*, providing important insights into plant secondary metabolism and valuable targets for breeding specific flower colors.

## 1. Introduction

*Lycoris*, a genus within the Amaryllidaceae family, holds significant value in global ornamental horticulture. These plants are prized for their unique architectural forms and a remarkable diversity of flower colors, ranging from pure white, yellow, and pink to vibrant purple and red [1,2]. This wide array of natural color variations not only provides an excellent resource for horticultural breeding but also offers a prime natural system for exploring the underlying molecular mechanisms of flower coloration [3]. However, detailed studies on the metabolic and genetic networks controlling *Lycoris* flower color remain limited, especially the nuanced molecular distinctions between different color types, such as white and blue-purple flowers.

The vibrant hues of angiosperm flowers are primarily determined by specialized plant compounds called flavonoids [4,5,6]. Among these, anthocyanins are the main pigments responsible for red, purple, and blue colors. The specific type of anthocyanin (e.g., cyanidin, delphinidin, pelargonidin) and its chemical modifications (like glycosylation, methylation, or acylation) directly influence the final color shade and intensity [7,8,9,10]. Other flavonoids, such as flavonols and flavones, often appear colorless or faintly yellow but play a crucial supportive role as co-pigments. They interact with anthocyanins to form stable molecular complexes, adjust vacuolar pH, or chelate metal ions, thereby influencing anthocyanin absorption, stability, and overall hue, particularly in maintaining blue or purple coloration [11]. The biosynthesis of these flavonoids follows a highly conserved pathway. It starts with the phenylpropanoid pathway and proceeds through a series of key enzymes, including chalcone synthase (CHS), chalcone isomerase (CHI), flavanone 3-hydroxylase (F3H), flavonoid 3′-hydroxylase (F3′H), flavonoid 3′,5′-hydroxylase (F3′5′H), dihydroflavonol 4-reductase (DFR), and anthocyanidin synthase (ANS) [12,13,14]. The activity levels of these enzymes, their specific gene expression, and competition for substrates are central to defining the ultimate flower color.

Interestingly, kaempferol and its glycosylated forms, which are types of flavonols, are frequently implicated in flower color regulation across various plant species [15]. For example, in *Meconopsis integrifolia*, increased expression of *flavonol synthase 2* (*FLS2*) leads to a higher accumulation of kaempferol derivatives, which act as key pigments to form stable yellow flowers [16]. Similarly, In *Corydalis ambigua*, blue petal coloration relies on a specific complex formed by cyanidin 3-*O*-sambubioside, ferric ions (Fe^3+^), and crucially, kaempferol 3-*O*-sambubioside. Reconstruction experiments demonstrated that while cyanidin provides the pigment base, the stable blue hue (with characteristic spectral properties) cannot be achieved without the essential participation of the flavonol kaempferol, which acts as a vital co-pigment alongside Fe^3+^ [17]. While kaempferol itself is usually light-colored or colorless, its presence can significantly modify the final visual outcome of anthocyanin-based colors [11,18]. Furthermore, the synthesis of kaempferol competes with anthocyanin synthesis for common precursors, meaning the way metabolic flow is directed can profoundly alter the final pigment composition.

Beyond the enzymes directly involved in biosynthesis, the entire flavonoid pathway is finely tuned by various transcription factors (TFs) [19,20]. The MYB-bHLH-WD40 (MBW) complex is recognized as a central regulatory module [21,22]. MYB transcription factors directly bind to specific DNA sequences (cis-elements) in the promoters of flavonoid pathway genes, either activating or repressing their transcription. In *Freesia hybrida*, the transcriptional repressors FhACE1 and FhAP2 interact with MYB proteins. This interaction dose-dependently weakens MYB proteins’ ability to bind to target gene promoters, thus negatively regulating the biosynthesis of pigments (like anthocyanins) [21]. Similarly, SoMYB44, by interacting with SobHLH130, triggers the expression of *SoUFGT1* in *Syringa oblata*, which then promotes anthocyanin accumulation [23]. Moreover, other TF families, such as bZIP, WRKY, NAC, and MADS-box, have also been reported to influence the synthesis of specific anthocyanins or flavonols [24,25,26,27]. Despite these general insights, the precise composition, specific roles, and regulatory patterns of these TF families in different *Lycoris* flower colors remain largely unexplored.

In recent years, long non-coding RNAs (lncRNAs), defined as RNA molecules over 200 nucleotides long that do not code for proteins, have emerged as crucial regulators in various aspects of plant growth, development, and secondary metabolism. LncRNAs can modulate gene expression through diverse mechanisms, including acting in *cis* (affecting nearby genes) or *trans* (affecting distant genes), serving as competing endogenous RNAs (ceRNAs) to sponge miRNAs, guiding chromatin remodeling complexes, or acting as molecular scaffolds [28,29]. In several common plants, lncRNAs have been shown to form intricate regulatory networks with miRNAs or mRNAs, thereby fine-tuning the expression of flavonoid pathway genes and related TFs, ultimately impacting color [30,31]. In apple, *MdLNC499* acts as a *cis*-regulator of *MdERF109* expression. Overexpressing or silencing *MdLNC499* in apple fruit and callus increases *MdERF109* transcription, which then promotes anthocyanin accumulation [32]. Similarly, in apple under low nitrogen stress, *LNC159c* inhibits *miR159c* expression. This indirectly frees *MsMYB10* from repression, leading to more anthocyanin [33]. However, the identification, functional prediction, and mechanistic links of lncRNAs with flavonoid metabolism and TF networks in *Lycoris* flower coloration are currently unknown.

Beyond the striking differences between white and blue-purple petals, understanding the metabolic or genetic divergence between blue-purple and red flower colors is also critical. These subtle variations within colored flowers are often dictated by the differential expression of key hydroxylase genes, such as *F3′H* and *F3′5′H*, which determine the type of anthocyanin backbone produced [34]. In *Pericallis hybrida*, delphinidin (a blue-purple pigment) synthesis is primarily driven by high expression of *F3′5′H* [10], while *F3′H* directs the synthesis of cyanidin (a red-purple pigment) in *Cymbidium lowianum* [35]. Therefore, this study aims to systematically analyze the differences in flavonoid metabolites (particularly kaempferol derivatives), key structural genes, transcription factors, and lncRNAs between white and blue-purple *Lycoris* petals by integrating lncRNA sequencing, mRNA transcriptome sequencing, and targeted metabolomics. Our goal is to construct a comprehensive lncRNA–mRNA–metabolite co-regulatory network to elucidate the molecular mechanisms behind blue-purple coloration and to explore the metabolic divergence between blue-purple and red petals. This research will provide systematic evidence for understanding the complex regulatory networks governing *Lycoris* flower color and offer theoretical foundations and candidate gene resources for molecular breeding and color modification in *Lycoris* and related ornamental plants.

## 2. Results

### 2.1. Analysis of Flower Color Traits and Differential Metabolites in L. longituba and L. sprengeri

**Figure 1:**
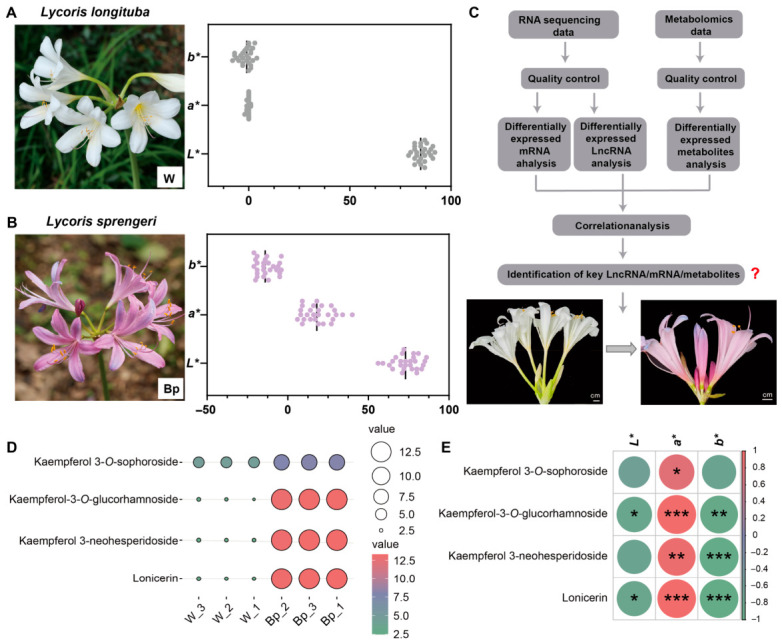
Overview of *Lycoris* flower phenotypes, analytical workflow, and key flavonoid metabolites. (**A**) Picture of *L. longituba* (W), showing its white flowers. The small graph next to it displays the CIELAB color readings for W petals. (**B**) Picture of *L. sprengeri* (Bp), showing its blue-purple flowers. The small graph next to it shows the CIELAB color readings for Bp petals. (**C**) Diagram showing our research steps. We used lncRNA sequencing and metabolomics data. Data quality was assessed, and differentially expressed lncRNAs (DELs) and differentially expressed metabolites (DEMs) were identified. Then, we linked lncRNAs, mRNAs, and metabolites to find important patterns. (**D**) This panel illustrates the differing levels of key kaempferol derivatives and related flavonoid metabolites. These differences are seen between white (W) and blue-purple (Bp) *Lycoris* petals. (**E**) This panel presents the correlation between four specific metabolites (Kaempferol-3-*O*-sophoroside, Kaempferol-3-*O*-glucorhamnoside, Kaempferol-3-*O*-neohesperidoside, and Lonicerin) and the CIELAB color parameters. Statistical significance levels are indicated by asterisks: * *p* < 0.05, ** *p* < 0.01, *** *p* < 0.001.

To clarify the nature of flower color differences between *Lycoris* species, we selected two representative types cultivated at the Hangzhou Botanical Garden for phenotypic analysis: the white-flowered *L*. *longituba* (Group W) and *L*. *sprengeri* (Group Bp) (Figure 1A,B). To objectively define the color of *L*. *sprengeri*, we measured the CIELAB parameters of the perianth using a colorimeter. The results showed that for Group W, both the a* values (red/green axis) and b* values (yellow/blue axis) were distributed near zero, while the L* values (lightness) were high, consistent with the high reflectivity of white petals. In contrast, the chromaticity distribution of Group Bp was distinctly different. Its a* values were positive (ranging from 6 to 40), indicating a significant red tone, while its b* values were negative (ranging from −4 to −21), indicating a clear blue component. The combination of a* > 0 and b* < 0 corresponds to a purple or blue-purple color perceived by the human eye, rather than pure-blue (where a* is near 0 and b* is significantly negative). Therefore, based on both objective color parameters and visual observation, the flower color of *L*. *sprengeri* is best described as “bluish purple.” In the subsequent results and discussion, we refer to the *L*. *sprengeri* samples as “blue-purple” (Bp).

To reveal the molecular differences between white and blue-purple petals, we integrated transcriptomic and metabolomic data for correlation analysis (Figure 1C). Targeted metabolomic data showed that various glycoside derivatives of the kaempferol family differed significantly between the W and Bp groups (Figure 1D, Table S1). Specifically, Kaempferol 3-*O*-sophoroside, Kaempferol-3-*O*-glucorhamnoside, and Kaempferol 3-neohesperidoside were highly enriched in blue-purple petals (Bp) but were undetectable or present in very low amounts in white petals (W). Additionally, a flavonoid compound called Lonicerin showed a similar accumulation pattern. Further Pearson correlation analysis (Figure 1E) confirmed that the levels of these four key metabolites were highly correlated with flower color parameters: they showed a significant positive correlation with the a* value representing red tones (*R* > 0.8, *p* < 0.001) and a significant negative correlation with the b* value representing blue tones (*R* < −0.8). Since a more negative b* value indicates a deeper blue color, this result strongly suggests that the high accumulation of kaempferol derivatives not only accompanies pigment synthesis but may also directly promote the formation of the bluish-purple hue in *L*. *sprengeri* through copigmentation.

### 2.2. Transcriptomic Analysis Reveals the Molecular Basis of Blue-Purple Flower Formation

**Figure 2:**
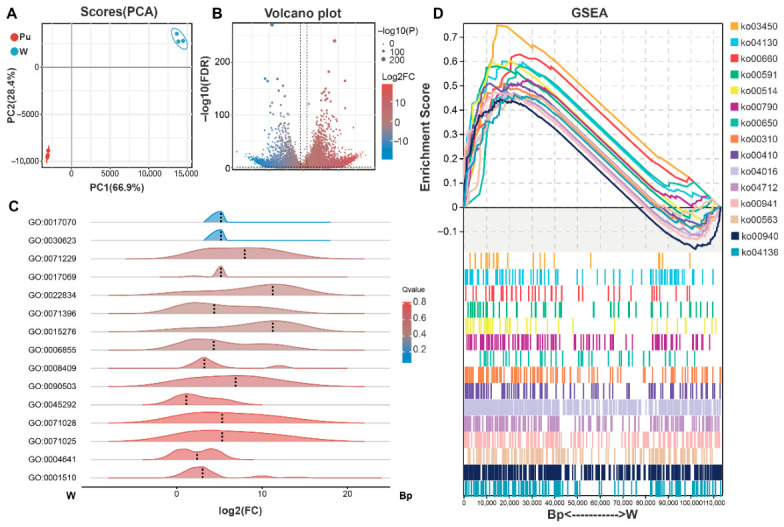
Gene activity differences in *Lycoris* petals. (**A**) PCA plot for gene expression. Each dot is a sample. The groups are clearly separate. PC1 (66.9%) and PC2 (28.4%) show most of the differences. The solid lines represent the axes of PC1 and PC2 (with 0 as the baseline), and the blue ellipse indicates the cluster of W group samples for visual differentiation. (**B**) Volcano plot showing genes that are more or less active. The *x*-axis shows how much gene activity changed (log2FC). The *y*-axis shows how sure we are about the change (−log10(FDR)). The vertical dashed line indicates the threshold of |Log2FC| = 1, and the horizontal dashed line represents the threshold of −log10(P) = 1.3 (*p* < 0.05). Red dots mean more active in Bp, blue dots mean less active. (**C**) Ridge plot showing what jobs these different genes do (GO terms). (**D**) GSEA plot for KEGG pathways. The top part shows that pathways for making colors are much more active in Bp flowers (NES > 0 means more active). The bottom part shows where the genes are in the overall list.

To deeply investigate the transcriptional regulatory mechanisms driving the color difference between *L*. *longituba* and *L*. *sprengeri*, we constructed high-quality cDNA libraries and performed RNA-seq sequencing (Table S2). Sequencing reads from all samples were successfully mapped to the reference genome, with total mapping rates (Total_Mapped%) exhibiting noticeable variations among different varieties. Overall, the data quality meets the requirements for downstream transcriptome analysis (Table S3). PCA results (Figure 2A) showed high clustering among biological replicates within the same group, indicating that the sequencing data was highly reproducible and reliable. We identified differentially expressed genes (DEGs) between W and Bp. The volcano plot (Figure 2B) visually displays the overall pattern of gene expression changes. Compared to W, a large number of genes in Bp were significantly up-regulated (red dots), while a considerable number were significantly down-regulated (blue dots). This large-scale transcriptomic change implies that the formation of the blue-purple phenotype is accompanied by complex remodeling of gene expression networks.

To analyze the biological functions of the DEGs, we performed GO enrichment analysis (Figure 2C). The ridge plot showed that differential genes were significantly enriched in multiple terms related to secondary metabolism and transcriptional regulation. Notably, the most significantly enriched terms were primarily associated with RNA splicing, degradation, surveillance, and methylation processes. Their log2(FC) distribution exhibited a clear positive shift, indicating upregulation in the blue-purple group. This finding suggests that post-transcriptional regulation, specifically mRNA alternative splicing and stability control, may also be involved in the regulation of blue-purple petal color formation, rather than merely relying on transcriptional activation. Furthermore, terms linked to ‘transcription regulatory factor activity’ were also significantly enriched, which implies a potential central role of transcription factors in regulating flower color. Pathway-level analysis using Gene Set Enrichment Analysis (GSEA) (Figure 2D) further supported these observations. It revealed high positive enrichment scores within the Bp group for pathways such as ‘Flavonoid Biosynthesis’ (ko00941), ‘Phenylpropanoid Biosynthesis’ (ko00940), and ‘Flavone and Flavonol Biosynthesis’ (ko00944). This aligns well with the previously reported high enrichment of kaempferol derivatives in the Bp group.

### 2.3. Transcriptional Reprogramming of the Flavonoid Biosynthetic Pathway Drives Metabolic Flux Divergence in Lycoris Flower Color

**Figure 3:**
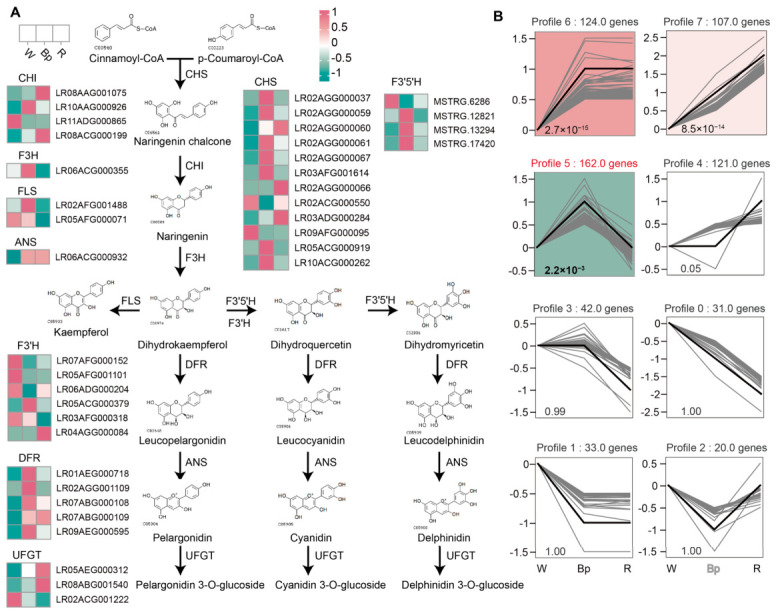
Gene activity in color-making pathway across different *Lycoris* Colors. (**A**) Diagram of the color-making pathway (flavonoid biosynthesis). Arrows show steps. Small boxes next to each step show how active the genes are in W (white), Bp (blue-purple), and R (red) flowers. Pink means very active. (**B**) Graph showing gene activity patterns (STEM clustering). This shows how gene activity changes from W to Bp to R. Profile 5 (162 genes) shows genes most active in Bp. Profile 4 (121 genes) shows genes most active in R. Profiles 6 and 7 show genes that become more active in both Bp and R. The black line is the average pattern for the group. The colored trend blocks represent significantly enriched trends, and different colors indicate distinct trends.

To precisely dissect the molecular mechanisms behind the color differences in *L*. *longituba* and *L*. *sprengeri*, and to compare metabolic differences with *L*. *radiata* var. *pumila* (red, R Figure S1A), we reconstructed the flavonoid biosynthetic pathway and analyzed the expression patterns of key structural genes in petals of the three color types (Figure 3A). Across all four metabolites presented in Figure 1, concentrations in red petals were significantly lower compared to blue-purple petals. This observation further supports the involvement of these four metabolites in promoting blue-purple coloration (Figure S1B). Compared to white petals, the expression patterns of upstream core genes showed a general upward trend in colored petals (Bp and R). For instance, *Chalcone Synthase* (*LrCHS*, multiple members of the CHS family) and *Anthocyanidin Synthase gene* (*LrANS*) showed higher expression levels in both Bp and R groups than in the W group. At the key metabolic node of dihydroflavonols, we observed a distinct divergence in metabolic flux. Initial transcriptomic analysis indicated the presence of several *Flavonoid 3′,5′*-*hydroxylase*-associated transcripts (*LrF3′5′H*, including entries such as MSTRG.12821, MSTRG.13294, and MSTRG.17420). To precisely define these candidate genes, we employed minimap2 to map sequences to the reference genome. Subsequently, utilizing transcriptome data from BAM files as evidence, we meticulously examined the transcriptional status of each locus with IGV. Ultimately, we confirmed four independent *F3′5′H* genes, each supported by robust transcriptional evidence. Three validated *F3′5′H* genes exhibited extremely high expression in blue-purple (Bp) petals, while their expression levels were very low in white (W) and red (R) petals. Profile 5 (162 genes) in the trend analysis (Figure 3B, Table S4) perfectly illustrated this feature; gene expression was low in W, peaked in Bp, and then dropped rapidly in R. This indicates that *LrF3′5′H* plays a decisive role in the formation of blue-purple petals by guiding metabolic flux toward dihydromyricetin, and subsequently synthesizing delphinidin (the precursor for blue/purple anthocyanins).

On the other hand, *Flavonol Synthase* (*LrFLS*, such as LR02AFG001488) genes were also significantly highly expressed in the Bp group, while expression was lower in the W and R groups. This is consistent with our earlier metabolomic finding that kaempferol derivatives are highly enriched in blue-purple petals (Figure 1). The high expression of *LrFLS* can promote the synthesis of flavonols (such as kaempferol), which serve as important copigments that work with delphinidin to give *L*. *sprengeri* its unique blue-purple tone. In sharp contrast to the blue-purple petals, the formation of red (R) petals mainly relied on the dominant expression of *Flavonoid 3′-hydroxylase* (*LrF3′H*). Multiple *LrF3′H* genes (such as LR06ADG000204, LR04AGG000084) showed higher expression levels in the R group, but were significantly repressed in the Bp group. This indicates that *LrF3′H* directs metabolic flux towards dihydroquercetin, which then leads to the synthesis of cyanidin (a red anthocyanin precursor), thus resulting in the formation of red petals. Therefore, we hypothesize that the diversity of *Lycoris* flower color is shaped by the metabolic flux diversion determined by the differential expression of key hydroxylase genes (*LrF3′5′H* and *LrF3′H*), alongside the synergistic action of *LrFLS*. The formation of blue-purple petals may depend on the co-activation of *LrF3′5′H* and *LrFLS*, whereas red petals are presumably driven primarily by the dominant expression of *LrF3′H*.

To clearly differentiate the contributions of gene expression from genetic background, we performed correlation analyses between these genes, the phenotype, and the four metabolites presented in Figure 1 (Tables S5 and S6). We observed a strong statistical correlation between the expression patterns of key flavonoid enzyme genes and flower color measurements (Lab values). Specifically, genes such as LR09AFG000095, LR11ADG000865, LR05AFG001101, and MSTRG.6286 exhibited extremely strong positive correlations in white (W) and blue-purple (Bp) varieties, while their correlation with red (R) varieties significantly decreased. This suggests a functional association between the expression levels of these genes and the coloration characteristics of white and blue-purple petals. Conversely, other genes (e.g., LR02AGG00060, LR07ABG000109, LR06ACG000932, LR05AEG000312) showed extremely high negative correlations in W and Bp varieties, with lower correlations in R varieties, indicating their potential involvement in flower color formation through inhibitory or negative regulatory mechanisms. This specific expression pattern, highly consistent with the gradient changes in flower color phenotypes, provides preliminary evidence that gene expression plays a dominant role in determining flower color. Furthermore, we observed that many genes (e.g., LR02AGG00037, LR05ACG000919, MSTRG.12821, MSTRG.17420, LR02AFG001488) showed extremely high positive correlations between their expression and the accumulation levels of the four metabolites (Kaempferol 3-*O*-sophoroside, Kaempferol-3-*O*-glucorhamnoside, Kaempferol 3-neohesperidoside, and Lonicerin) presented in Figure 1. This tight correlation between gene expression and metabolite accumulation directly reveals the clear functional role of these genes in regulating the formation of specific flavonoid metabolites within the biochemical pathway. Thus, despite genetic background differences among *Lycoris* varieties with distinct flower colors, our integration of transcriptome and metabolome data, and the establishment of strong correlative links between gene expression and specific biochemical products, support that these genetic background differences reshape flavonoid metabolic flux through the differential expression of specific key genes, thereby determining the final flower color phenotype, rather than merely non-specific effects attributable to a general genetic background.

### 2.4. LrWRKY70 Promotes Flavonoid Biosynthesis and Influences Lycoris Flower Color Formation

**Figure 4:**
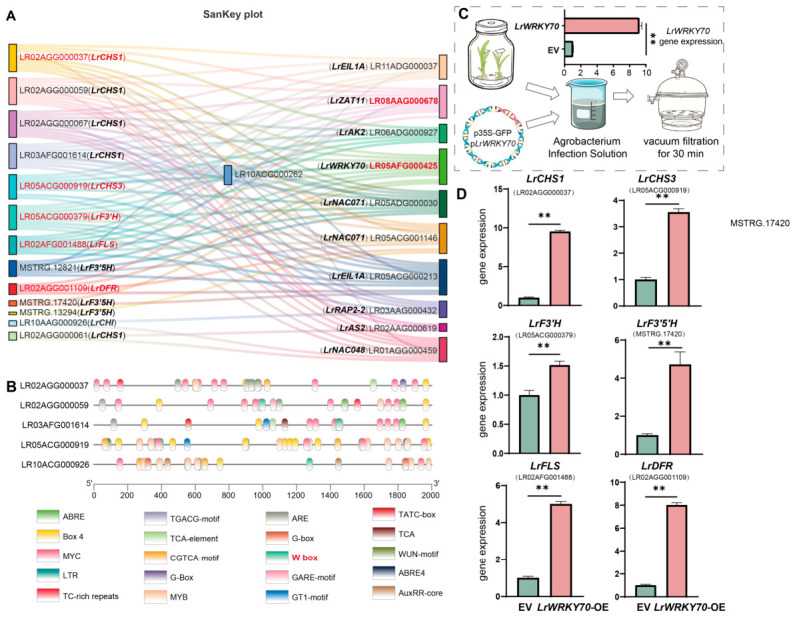
Network analysis and functional validation of LrWRKY70 in *Lycoris* flower color regulation. (**A**) Sankey diagram showing links between color-making genes (left) and genes that control them (transcription factors, right). Lines show strong connections (Pearson correlation > 0.9). *LrWRKY70* (in red) and *LrZAT11* have many connections, suggesting they are important. (**B**) Diagram showing control signals (cis-acting elements) on the DNA near color-making genes. These signals tell other genes what to do. The W-box (in red) is a special signal for WRKY genes like *LrWRKY70*. Its presence suggests LrWRKY70 can control these genes. (**C**) Picture showing how we tested *LrWRKY70*. We put the *LrWRKY70* gene into *Lycoris* seedlings using bacteria. We used a vacuum for 30 min to help the gene get in. (**D**) Relative expression determined by qRT-PCR. Bar graphs showing how active the color-making genes were after we added extra *LrWRKY70* (labeled *LrWRKY70*-OE). Compared to regular plants (EV), many color-making genes (like *LrCHS1*, *LrCHS3*, *LrF3′H*, *LrF3′5′H*, *LrFLS*, *LrDFR*) became much more active. Stars mean the change was very clear (** *p* < 0.01). This confirms LrWRKY70 helps make colors. Values are mean ± SD of three biological replicates (each with three technical replicates). Expression was normalized to ACTIN and calculated by the 2^−ΔΔCt^ method. Statistical significance according to Student’s *t*-test: ** *p* < 0.01.

We used the transcription factors (TFs) identified in Profile 5 of the trend analysis above to perform a co-expression network analysis with structural enzyme genes in the flavonoid biosynthetic pathway. We selected TFs with a Pearson correlation coefficient greater than 0.9 and constructed a Sankey plot showing their high correlation with flavonoid structural genes (Figure 4A, Table S7). The results revealed complex many-to-many regulatory relationships between multiple transcription factors and structural genes. Among these candidate transcription factors, *LrWRKY70* (LR05AFG000425) and *LrZAT11* (LR08AAG000678) showed the highest degree of connectivity, meaning they had strong positive correlations with the largest number of flavonoid structural genes (including *LrCHS1*, *LrF3′H*, *LrF3′5′H*, *LrFLS*, *LrDFR*, etc., covering key enzymes upstream, midstream, and downstream). This suggests these two transcription factors might be core hubs regulating *Lycoris* flower color formation. We further analyzed the cis-acting elements in the 2000 bp promoter regions upstream of several flavonoid structural genes (such as *LrCHS1*, *LrCHS3*, *LrF3′H*, *LrFLS*, *LrDFR*, etc.) (Figure 4B). The results showed that these promoter regions are rich in various known regulatory elements, including light-responsive elements (like Box 4, G-box), hormone-responsive elements (like ABRE), and MYB binding sites. More critically, we found that W-boxes (specific binding sites for WRKY transcription factors) are commonly present in the promoter regions of these genes. Given the widespread role of WRKY transcription factors in regulating plant secondary metabolism, we prioritized *LrWRKY70* for further study.

To validate the role of LrWRKY70 in *Lycoris* flower color regulation, an overexpression vector (p35S::LrWRKY70) containing the *LrWRKY70* coding sequence was constructed. This vector was then successfully introduced into *Lycoris* tissue culture seedlings through *Agrobacterium*-mediated vacuum infiltration (Figure 4C). Subsequently, we selected the genes with the greatest differences in flavonoid structural genes between white and blue-purple petals for subsequent validation. We used qRT-PCR (quantitative real-time PCR) to detect their expression changes (Figure 4D). The results showed that compared to the control group transformed with the empty vector (EV), the expression levels of multiple key flavonoid structural genes were significantly upregulated in seedlings overexpressing *LrWRKY70*. These genes covered the upstream (e.g., *LrCHS1*, *LrCHS3*), midstream (e.g., *LrF3′H*, *LrF3′5′H*, *LrFLS*), and downstream (e.g., *LrDFR*) parts of the pathway. For example, *LrCHS1* expression increased nearly 9-fold, and *LrFLS* and *LrDFR* expression also rose significantly by about 4–8 times. At the same time, the expression of genes determining anthocyanin type, such as *LrF3′H* and *LrF3′5′H*, was also significantly upregulated. This regulatory effect is likely achieved by LrWRKY70 recognizing W-box elements in the promoters of downstream genes, thereby promoting the accumulation of anthocyanins and flavonols, and ultimately influencing *Lycoris* flower color formation.

### 2.5. Distal Cis-Acting lncRNA lncRNA401 Mediates Positive Regulation of Flavonoid Biosynthesis by LrWRKY70

**Figure 5:**
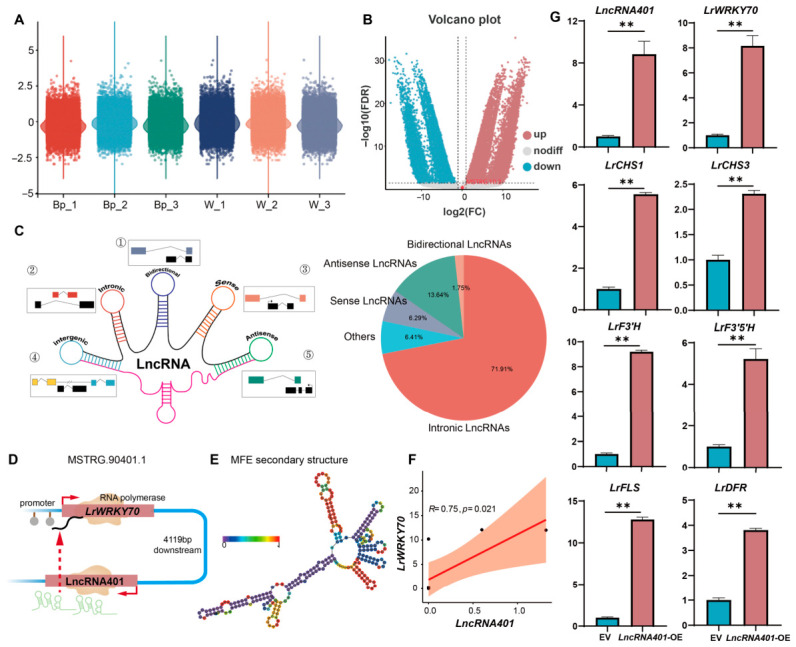
*LncRNA401* controls *LrWRKY70* to make blue-purple flowers. (**A**) Violin plots showing how much all lncRNAs are active in blue-purple (Bp) and white (W) flower samples. Each sample has 3 repeats. (**B**) Volcano plot showing which lncRNAs are more or less active between blue-purple (Bp) and white (W) flowers. The *x*-axis represents log2(fold change Bp vs. W), with positive values indicating upregulation in Bp. The *y*-axis represents −log10(adjusted *p*-value). Red dots indicate genes significantly upregulated in Bp; blue dots indicate genes significantly downregulated in Bp; gray dots are not significant. Significance thresholds: |log2FC| > X and FDR < 0.05. (**C**) Pie chart showing different types of lncRNAs based on where they are in the plant’s DNA. Most lncRNAs are in the middle of other genes (intronic). The dashed lines in the schematic indicate the intronic regions of the corresponding genes/transcripts, while the solid rectangles represent exonic regions. The numbers ①–⑤ in the schematic diagram correspond to the five categories of lncRNAs (bidi rectional, intronic, sense, intergenic, and antisense), which are also quantified in the pie chart on the right. (**D**) The schematic diagram shows the location of *LncRNA401* near the *LrWRKY70* gene. *LncRNA401* is situated downstream of *LrWRKY70* and is transcribed from the antisense strand of the gene (approximately 4119 base pairs apart). (**E**) Picture of *LncRNA401*’s predicted 3D shape (secondary structure). This shape is important for how it works. The colors show how stable different parts of the shape are. (**F**) Graph showing the relationship between *LncRNA401* activity and *LrWRKY70* activity. There is a strong positive link (R = 0.75, *p* = 0.021). This means when LncRNA401 is more active, *LrWRKY70* is also more active. The red solid line represents the linear regression fit between LncRNA401 and LrWRKY70 expression levels, and the orange shaded area indicates the 95% confidence interval of the regression. (**G**) Relative expression measured by qRT-PCR. Bar graphs showing gene activity. We added extra *LncRNA401* into plants. This made *LncRNA401* itself much more active. It also made *LrWRKY70* much more active. And many color-making genes (like *LrCHS1*, *LrCHS3*, *LrF3′H*, *LrF3′5′H*, *LrFLS*, *LrDFR*) also became much more active. This shows that *LncRNA401* helps *LrWRKY70*, which then helps make colors. Values are mean ± SD of three biological replicates (each with three technical replicates). Expression was normalized to ACTIN and calculated by the 2^−ΔΔCt^ method. Statistical significance according to Student’s *t*-test: ** *p* < 0.01.

Our previous analysis revealed the overall activation of the flavonoid biosynthetic pathway in blue-purple (Bp) petals and identified LrWRKY70 as a core transcription factor. To further explore the regulatory mechanisms upstream of *LrWRKY70*, particularly the involvement of lncRNAs, we first performed comprehensive identification and differential expression analysis of lncRNAs in white (W) and blue-purple (Bp) *Lycoris* petals. RNA-seq data showed that the expression distribution of lncRNAs across different samples was consistent (Figure 5A). Through differential expression analysis (Figure 5B), we identified a large number of differentially expressed lncRNAs (DELs) between Bp and W. These lncRNAs were mainly classified as intronic lncRNAs (71.91%), antisense lncRNAs (13.64%), and intergenic lncRNAs (6.29%) (Figure 5C). Based on these DELs, through genomic location analysis and cis-action prediction, we screened an lncRNA that has a cis-relationship with the *LrWRKY70* coding gene (MSTRG.90401.1) and named it *LncRNA401*. This lncRNA is located approximately 4119 bp downstream of the *LrWRKY70* antisense strand (Figure 5D). This close collinear arrangement suggests that *LncRNA401* may regulate *LrWRKY70* expression via a cis-acting mechanism.

To assess the potential functional properties of *LncRNA401*, we predicted its secondary structure. Results showed that *LncRNA401* can fold into a complex stem-loop structure with a predicted minimum free energy (MFE) of −85.7 kcal/mol (Figure 5E). Subsequently, we analyzed the expression correlation between *LncRNA401* and *LrWRKY70* in all samples. The results indicated a significant positive correlation between the expression levels of *LncRNA401* and *LrWRKY70* (Pearson R = 0.75, *p* = 0.021, Figure 5F), further supporting a potential positive regulatory role of *LncRNA401* on *LrWRKY70*.

To experimentally verify the function of *LncRNA401*, we used the Agrobacterium-mediated transient transformation system to overexpress *LncRNA401* in *Lycoris* tissue culture seedlings (Figure 4C, same method). qRT-PCR results (Figure 5G) showed that compared to the empty vector (EV) control, overexpression of *LncRNA401* significantly promoted the transcription of the *LrWRKY70* gene, increasing its expression by about 9-fold. Further testing of the effect of *LncRNA401* overexpression on downstream flavonoid structural genes showed that overexpression of *LncRNA401* similarly activated the expression of multiple key flavonoid structural genes. The upregulation fold changes in these genes were highly consistent with the results observed when *LrWRKY70* was overexpressed (Figure 4D).

**Figure 6:**
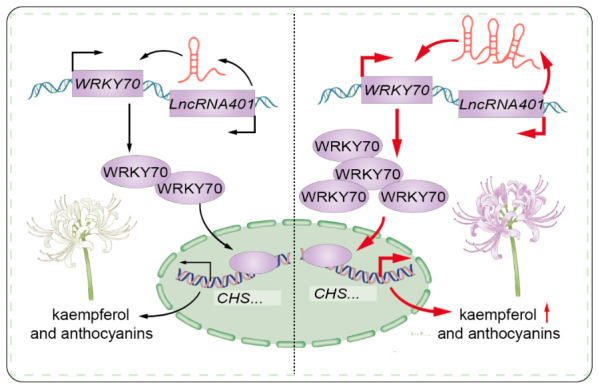
Proposed working model illustrating the regulatory mechanism of *LncRNA401* in *Lycoris* flower color formation. Arrows indicate the direction of transcriptional activation, protein expression, and metabolic flux. In white-flowered *Lycoris* (left), low expression of *LncRNA401* results in basal *WRKY70* expression, leading to moderate accumulation of kaempferol and anthocyanins. In blue-purple *Lycoris* (right), high expression of *LncRNA401* upregulates *WRKY70* transcription, which in turn promotes the expression of anthocyanin biosynthesis genes (e.g., *CHS*) and significantly increases the accumulation of kaempferol and anthocyanins, resulting in deepened flower color.

Finally, Figure 6 presents a concise molecular mechanism model summarizing the core findings of this study: the long non-coding RNA (*LncRNA401*) may positively regulate the expression of the transcription factor LrWRKY70, which in turn may activate the downstream flavonoid biosynthetic pathway. This could potentially lead to the accumulation of anthocyanins and kaempferol derivatives in the petals, which may influence the presentation of flower color. This model suggests a potential hierarchical regulatory network involving *LncRNA401*–*LrWRKY70*—Structural Genes (e.g., *LrCHS*)—Secondary Metabolites (Kaempferol/Anthocyanins)—Flower Color Phenotype. *LncRNA401* might act as a potential molecular switch, which may amplify the flavonoid synthesis signal by upregulating *LrWRKY70* expression, and thus may be involved in the regulatory process of *Lycoris* petal coloration, though the specific regulatory mechanism requires further verification by biochemical experiments.

## 3. Discussion

True “blue” flowers, like cornflowers and Himalayan blue poppies, are relatively rare in nature, and their formation mechanisms are often quite complex. Our color difference data for *L*. *sprengeri* clearly placed its flower color within the ‘blue-purple’ category (defined by a* and b* values), a finding consistent with previous studies [36]. It is widely recognized that most flowers perceived as blue are, in reality, blue-purple transitional shades, created by the combined action of anthocyanins (predominantly delphinidin or cyanidin) and co-pigments [37]. Notably, our metabolomic results revealed that the most significant metabolic difference distinguishing white from blue-purple *Lycoris* is not anthocyanins themselves, but rather kaempferol glycosides. Typically, anthocyanins are considered the direct source of color, while flavonols (like kaempferol) are pale yellow or colorless. However, our results fit well with the theory of “copigmentation.” Existing studies show that flavonols, acting as typical copigments, can form stable complexes with anthocyanins through molecular stacking. This interaction not only stabilizes the flower color but also produces a bathochromic shift, moving the color toward longer wavelengths in the blue spectrum [11,38]. Therefore, we speculate that in *L*. *sprengeri*, the high abundance of kaempferol derivatives does not serve as a “background color,” but rather acts as a key driver of the blue shift, working synergistically with anthocyanins to create its unique blue-purple hue. This explains why these colorless flavonols are lacking in white species but are extremely abundant in colored ones.

By integrating transcriptomic analyses of white, blue-purple, and red *Lycoris* petals, the study systematically uncovers the molecular mechanisms governing flower color diversity in the *Lycoris* genus, with a specific focus on the key metabolic branching points that distinguish "white" from "colored" phenotypes and "blue-purple" from "red" phenotypes. Figure 3A clearly shows the expression patterns of key structural genes in the flavonoid biosynthetic pathway, confirming that dihydroflavonols are the core branching node for color differentiation. We speculate that the formation of blue-purple petals in *L*. *sprengeri* (Bp) mainly depends on the high expression of transcripts related to *LrF3′5′H*. These genes specifically direct metabolic flux toward the biosynthesis of delphinidin. With more hydroxyl groups on its B-ring, delphinidin serves as the primary basis for the formation of blue or purple anthocyanins [39]. Meanwhile, the high expression of the *LrFLS* gene in Bp petals may promote the massive accumulation of kaempferol derivatives, which is highly consistent with our previous metabolomic results (Figure 1). As an important copigment, kaempferol can form stable complexes with anthocyanins and effectively regulate vacuolar pH, thereby enhancing blue hues and improving color stability. Thus, the unique blue-purple color of *L*. *sprengeri* is constructed jointly by *LrF3′5′H*-led delphinidin synthesis and the copigmentation effect of kaempferol mediated by *LrFLS*. This synergistic mechanism of “pigment skeleton + copigment” has been reported in many blue-purple flowers (such as roses and hydrangea) [40,41,42], but our study provides evidence for its fine regulation at the transcriptional level in *Lycoris*. In contrast, the formation of red *L*. *radiata* var. *pumila* (R) petals is driven by the dominant expression of the *LrF3′H* gene. *LrF3′H* is responsible for hydroxylation at the 3′ position of the B-ring, directing metabolic flux toward cyanidin synthesis [43,44]. During this process, the expression of the *LrF3′5′H* gene is markedly suppressed in R petals, blocking delphinidin synthesis. This “either-or” competitive expression pattern between *LrF3′5′H* and *LrF3′H* is a classic mechanism for plant flower color differentiation between red and blue lineages. The inverse expression patterns shown in Profile 5 and Profile 4 of the trend analysis (high expression unique to Bp vs. high expression unique to R) precisely depict the transcriptomic basis of this metabolic divergence. Of course, given the differences in genetic backgrounds, we also performed correlation analyses between these genes, metabolites, and phenotypes to confirm that gene expression levels are the main cause of flower color variation. However, due to the lack of a pan-genome covering all *Lycoris* species, the current alignment against the single reference genome exhibits certain reference bias, which may lead to the loss of some transcripts or redundant assembly. Nevertheless, the core *F3′5′H* gene we identified still showed significantly higher expression in Bp. Our conclusion that transcript expression changes redirect metabolic flux therefore remains valid. In the future, further functional verification via target gene cloning or long-read sequencing will be required to strengthen these findings.

Our study also found that even within the same enzyme family (such as CHS or CHI), different members show highly heterogeneous expression patterns across different flower color types (Figure 3A). For example, some *LrCHS* genes are upregulated in both Bp and R, while others are specifically upregulated only in Bp. This phenomenon likely reflects subfunctionalization or neofunctionalization under gene redundancy, meaning different members of the same family may have evolved specific regulatory functions for different developmental stages, tissues, or color types [45]. For instance, a specific LrCHS homolog might play a more important role in blue-purple petals rather than just serving as a general upstream enzyme [46]. This internal heterogeneity adds complexity to the color regulatory network and suggests that future research could deeply explore the functional differentiation of these gene family members.

WRKY transcription factors are widely involved in stress response, growth, and secondary metabolism regulation in plants [47]. Our study discovered the central role of LrWRKY70 in *Lycoris* flower color formation, adding to the functional spectrum of the WRKY family in color regulation. In apples (*Malus domestica*), MdWRKY10 was found to promote anthocyanin accumulation [48], and in red pears, PyWRKY26 also plays a role in fruit red pigment synthesis [49]. The discovery of LrWRKY70 echoes these studies, highlighting the importance of the WRKY gene family in pathways outside the MYB-bHLH-WD40 complex. In the Sankey plot (Figure 4A), the zinc finger protein transcription factor LrZAT11 was also highly correlated with multiple structural genes. This suggests that *Lycoris* flower color regulation may not be carried out by a single transcription factor alone, but by a complex regulatory network involving the synergistic action of multiple transcription factors like WRKY and ZAT. These factors may work together through interactions, cascade regulation, or by responding to different upstream signals (such as hormones or light) to finely tune the flower color phenotype.

Even though *LncRNA401* lies about 4.1 kb downstream of the *LrWRKY70* gene, its overexpression markedly boosts *LrWRKY70* transcription (Figure 5G). This effect highlights *LncRNA401*’s ability to influence nearby gene activity. We also found a strong co-expression pattern between *LncRNA401* and *LrWRKY70* (Figure 5F). This strong, long-distance activation suggests *LncRNA401* might use an unusual *cis*-acting mechanism. For instance, it could behave like an enhancer RNA (eRNA) [50], remotely changing how accessible the chromatin is or the promoter activity at the *LrWRKY70* gene site. Another possibility is that *LncRNA401* acts as an RNA scaffold [51]. It might bind to the *LrWRKY70* mRNA, thereby boosting its stability or translation efficiency. Notably, *LncRNA401*’s predicted stable secondary structure (Figure 5E) likely provides a crucial structural basis for binding with proteins or other nucleic acids. This binding then helps mediate its long-distance control. Therefore, more in-depth experimental study is needed to fully understand the exact control link between *LncRNA401* and *LrWRKY70* and how it functions.

## 4. Materials and Methods

### 4.1. Plant Materials and Growth Conditions

For this study, three distinct *Lycoris* species with characteristic flower colors were collected from the Hangzhou Botanical Garden (N 30°15′34″, E 120°07′15″), which experiences a typical subtropical monsoon climate at an average altitude of 20 m. The white-flowered *L*. *longituba* (designated as W), the blue-purple-flowered *L*. *sprengeri* (designated as Bp), and the red-flowered *L*. *radiata* var. *pumila* (designated as R) were used. All plants were cultivated under standard horticultural conditions within the Hangzhou Botanical Garden.

For metabolomics analysis, RNA sequencing, and functional validation, fully opened flower petals (at the initial bloom stage, typically 3–5 days after bud opening) were collected. Three independent biological replicates were sampled for each flower color type (W, Bp, R), with each replicate consisting of petals from 3–5 individual flowers to account for biological variation. Immediately after collection, all samples were flash-frozen in liquid nitrogen and stored at −80 °C until further processing.

### 4.2. Flower Color Measurement

*Lycoris* flower color parameters were objectively measured using a Konica Minolta Chroma Meter CR-400 (Konica Minolta Inc., Tokyo, Japan) in the CIELAB color space. For each biological replicate, measurements were taken from three different points on the adaxial surface of three distinct petals from three individual flowers. The L* value represents lightness (0 = black, 100 = white), the a* value indicates the red-green chromaticity (+a = red, −a = green), and the b* value indicates the yellow-blue chromaticity (+b = yellow, −b = blue). The mean of these measurements was calculated for each sample.

### 4.3. Metabolomics Analysis

Approximately 100 mg of flash-frozen flower petals were ground into fine powder in liquid nitrogen. We added 1 mL of pre-chilled extraction solution (water/acetonitrile/isopropanol = 1:1:1, *v*/*v*/*v*), homogenized the mixture for 60 s, and extracted metabolites by ultrasonication at low temperature for 30 min. The sample was centrifuged at 12,000 rpm for 10 min at 4 °C, and the supernatant was stored at −20 °C for 1 h to precipitate proteins. We centrifuged the sample again at 12,000 rpm for 10 min at 4 °C, collected the supernatant, and dried it under vacuum. The residue was dissolved in 0.2 mL of 30% acetonitrile solution, homogenized, and centrifuged at 14,000 rpm for 15 min at 4 °C; the final supernatant was used for instrumental analysis. Chromatographic analysis was performed on a Vanquish UPLC system with a Waters HSS T3 column (Waters Corp., Milford, MA, USA, 50 × 2.1 mm, 1.8 μm), set at a column temperature of 40 °C, a flow rate of 0.3 mL/min, and an injection volume of 2 μL. Mobile phase A was water containing 0.1% acetic acid and mobile phase B was acetonitrile containing 0.1% acetic acid, with the gradient elution program as follows: 0–2.0 min (90% A, 10% B), 2.0–6.0 min (90% A to 40% A), 6.0–9.0 min (40% A, 60% B), 9.0–9.1 min (40% A to 90% A), and 9.1–12.0 min (90% A, 10% B). Mass spectrometry data were collected on a Q Exactive HFX mass spectrometer (Thermo Fisher Scientific, Waltham, MA, USA) with a heated ESI source using the Full-ms-ddMS2 acquisition mode; key parameters included sheath gas pressure of 40 arb, aux gas pressure of 10 arb, spray voltage of +3000 V and −2800 V, ion source temperature of 350 °C, ion transfer tube temperature of 320 °C, a full-scan mass range of m/z 70–1050 Da, and resolution of 70,000 for full MS and 17,500 for MS/MS. Raw data were acquired with Xcalibur 4.1 software (Thermo Fisher Scientific, https://www.thermofisher.com), then processed and quantified with TraceFinder™ 4.1 software (Thermo Fisher Scientific, https://www.thermofisher.com), and quantitative results were exported in Excel format; all steps were carried out at low temperature to maintain metabolite stability.

### 4.4. mRNA and lncRNA Sequencing

Total RNA was extracted from approximately 100 mg of flash-frozen flower petals using the TRIzol Reagent (Life Technologies, Carlsbad, CA, USA) according to the manufacturers’ instructions. RNA quality and quantity were assessed using a NanoDrop 2000 spectrophotometer (Thermo Fisher Scientific, Waltham, MA, USA) and an Agilent 2100 Bioanalyzer (Agilent Technologies, Santa Clara, CA, USA), with RNA integrity number (RIN) values typically above 7.0.

For mRNA sequencing, poly(A) RNA was enriched from 3 µg of total RNA using oligo(dT) magnetic beads (Vazyme Biotech Co., Ltd., Nanjing, China). For lncRNA sequencing, ribosomal RNA was removed using a Ribo-Zero rRNA Removal Kit (Vazyme Biotech Co., Ltd., Nanjing, China, Cat. #N409) to obtain total RNA containing lncRNA. Subsequently, mRNA and lncRNA sequencing libraries were prepared using the VAHTS Universal V6 RNA-seq Library Prep Kit for Illumina (Vazyme Biotech Co., Ltd., Nanjing, China, Cat. #NR604) following the manufacturer’s recommendations. Paired-end sequencing (150 bp reads) was performed on an Illumina NovaSeq 6000 platform (Illumina Inc., San Diego, CA, USA) by Guangzhou Gene Denovo Honour Biotechnology Co., Ltd. (Guangzhou, China).

### 4.5. De Novo Transcriptome Assembly and LncRNA Identification

Raw sequencing reads underwent initial quality filtering to remove adaptor sequences, low-quality reads (Phred score < 20), and reads containing undetermined bases (Ns). Clean reads were then mapped to this *L*. *radiata* var. *pumila* genome (unavailable to the public) assembly using HISAT2 [52] (version 2.2.1) with default parameters. Transcripts were subsequently reconstructed from the mapped reads in a genome-guided manner using StringTie [53] (version 2.1.4). These reconstructed transcripts were then subjected to functional annotation against public databases including NCBI non-redundant protein (Nr), Swiss-Prot, Gene Ontology (GO), Kyoto Encyclopedia of Genes and Genomes (KEGG), and Pfam using BLAST+ (version 2.13.0) (E-value < 1 × 10^−5^) to identify known protein-coding genes. For lncRNA identification, all reconstructed transcripts exceeding 200 nucleotides in length were rigorously filtered. Transcripts with known protein-coding potential were removed using a combination of CPC2 (Coding Potential Calculator 2; http://cpc2.cbi.pku.edu.cn) [54], CNCI (Coding-Non-Coding Index; https://github.com/www-bioinfo-org/CNCI (accessed on 13 January 2026)) [55], and Pfam protein domain analysis was performed using TransDecoder (https://github.com/TransDecoder/TransDecoder (accessed on 13 January 2026)). The remaining non-coding transcripts were then classified as putative lncRNAs. Their genomic classification (intergenic, intronic, sense-overlapping, antisense-overlapping, or bidirectional) was precisely determined by comparing their coordinates with annotated protein-coding gene models on the preliminary genome assembly using EDTools (version 2.29.2; https://bedtools.readthedocs.io) [56].

### 4.6. Bioinformatics Analysis

Read mapping and quantification: Clean reads were mapped to the de novo assembled transcriptome using HISAT2 (version 2.2.1). Gene expression levels were quantified as Fragments Per Kilobase of transcript per Million mapped reads (FPKM) using RSEM [57] (version 1.3.1). Identification of the F3′5′H gene: To precisely identify the F3′5′H gene locus, raw RNA-seq reads were aligned to the reference genome with Minimap2 (v2.30) [58]. The BAM-formatted alignment files provided direct evidence for inspecting transcriptional profiles. Each mapped locus was further visualized and verified using the Integrative Genomics Viewer (IGV, v 2.19.7) [59]. Differential Expression Analysis: Differentially expressed genes (DEGs) and lncRNAs (DELs) between different *Lycoris* samples (Bp vs. W, R vs. W, R vs. Bp) were identified using DESeq2 R package [60] (version 1.30.0). Genes with |log2 Fold Change| ≥ 1 and False Discovery Rate (FDR) < 0.05 were considered significantly differentially expressed. Functional Enrichment Analysis: Gene Ontology (GO) enrichment analysis was performed using the topGO R package. KEGG pathway enrichment analysis and Gene Set Enrichment Analysis (GSEA) were conducted using the clusterProfiler R package (version 3.18.1; https://bioconductor.org/packages/clusterProfiler (accessed on 13 January 2026)). Co-expression Network and Promoter Analysis: Pearson correlation coefficients between lncRNA, TF, and structural gene expression levels were calculated. Only correlations with |r| ≥ 0.9 and *p* < 0.05 were used to construct co-expression networks visualized by Sankey plot in R. For promoter analysis, 2000 bp upstream sequences of selected structural genes were extracted from the assembled transcriptome, and cis-acting elements were predicted using PlantCARE (http://bioinformatics.psb.ugent.be/webtools/plantcare/html/ (accessed on 13 January 2026)) and JASPAR databases (https://jaspar.genereg.net). LncRNA Structure Prediction: The secondary structure of identified lncRNAs was predicted using the RNAfold web server (http://rna.tbi.univie.ac.at/cgi-bin/RNAWebSuite/RNAfold.cgi (accessed on 13 January 2026)), with both Minimum Free Energy (MFE) and Centroid secondary structures considered. 

### 4.7. Transient Transformation and Functional Verification

For functional validation of candidate genes and lncRNAs, *Lycoris* tissue culture seedlings were used for *Agrobacterium*-mediated transient transformation. The coding sequence of *LrWRKY70* (or LncRNA401 cDNA sequence) was cloned into a modified pROKII vector under the control of the cauliflower mosaic virus (CaMV) 35S promoter, typically fused with Green Fluorescent Protein (GFP) for visualization (pROKII-35S::*LrWRKY70*-GFP or pROKII-35S::LncRNA401). Empty vector (EV) was used as a negative control.

*Agrobacterium tumefaciens* strain GV3101 carrying the recombinant plasmids was grown overnight in LB medium containing appropriate antibiotics. The bacterial cells were harvested by centrifugation, then resuspended in infiltration buffer (1/2 MS liquid medium, 10 mM MES pH 5.7, 200 µM AS) to an optical density (OD600) of 0.8–1.0. *Lycoris* seedlings were then submerged in the *Agrobacterium* suspension and subjected to vacuum infiltration for 30 min at 0.05 MPa. After infiltration, seedlings were blotted dry and incubated in a growth chamber (16 h light/8 h dark, 25 °C) for 3–5 days. Transformed tissues were then harvested for RNA extraction and qRT-PCR analysis.

### 4.8. RNA Extraction and RT-qPCR

To verify RNA-seq data and perform functional verification, total RNA was first extracted from transiently transformed *Lycoris* tissue-cultured plants. Petals were ground in liquid nitrogen, lysed thoroughly in TRIzol (Life Technologies, Carlsbad, CA, USA), and centrifuged to remove debris. After extraction with chloroform, the upper aqueous phase was collected and mixed with isopropanol to precipitate RNA. The RNA pellet was washed twice with 75% ethanol, air-dried, dissolved in RNase-free water, and quality-checked using NanoDrop (Thermo Fisher Scientific, Waltham, MA, USA) and agarose gel electrophoresis before storage at −80 °C. First-strand cDNA was synthesized from 1 µg of total RNA using the PrimeScript RT Reagent Kit with gDNA Eraser (TaKaRa Bio Inc., Shiga, Japan). Quantitative real-time PCR (qRT-PCR) was performed on an Applied Biosystems StepOnePlus Real-Time PCR System (Thermo Fisher Scientific, Waltham, MA, USA) using SYBR Premix Ex Taq II (TaKaRa Bio Inc., Shiga, Japan). Specific primers for target genes (lncRNAs, TFs, and structural genes) and the internal control gene *Lycoris* Actin were designed using Primer Premier 6.0 software (http://www.premierbiosoft.com/, Tables S8 and S9). The qRT-PCR reaction conditions were: 95 °C for 30 s, followed by 40 cycles of 95 °C for 5 s and 60 °C for 30 s. Relative gene expression levels were calculated using the 2^−ΔΔCt^ method [61], with three technical replicates for each biological sample.

### 4.9. Statistical Analysis

All data are presented as means ± standard deviation (SD). Statistical significance between two groups was determined using Student’s *t*-test. Pearson correlation coefficients were calculated to assess the relationships between gene expression levels and metabolite concentrations. A *p*-value less than 0.05 was considered statistically significant (*p* < 0.05), with increasing asterisks indicating higher levels of significance (*p* < 0.01 (**), *p* < 0.001 (***), *p <* 0.0001 (****)). All statistical analyses were performed using GraphPad Prism (version 10.0).

## 5. Conclusions

This study investigated the molecular basis of flower color in *Lycoris*. Through color measurements, we defined the distinctive bluish-purple hue of *L. sprengeri* petals. Metabolite analysis showed that specific kaempferol glycosides accumulate significantly in these petals and may act as key copigments that contribute to the blue-purple coloration. Transcriptome data revealed that different enzyme gene expression patterns drive color differences. High expression of *LrF3′5′H* and *LrFLS* in blue-purple petals likely directs metabolism toward delphinidin and kaempferol production, while high expression of *LrF3′H* in red petals likely leads to cyanidin accumulation. A key finding was the identification of LrWRKY70, a candidate transcription factor, and a novel long noncoding RNA called *LncRNA401*, which may upregulate *LrWRKY70* expression. This *LncRNA401*-LrWRKY70 module may then activate several downstream flavonoid biosynthesis genes. This regulatory network may explain how metabolic changes contribute to the unique blue-purple petal color of *Lycoris*, although the specific mechanism needs further biochemical validation. Our results suggest that lncRNAs and WRKY factors play important roles in plant pigmentation and could provide valuable targets for future *Lycoris* breeding programs aimed at developing specific flower colors.

## Data Availability

The genome and transcriptome data of *Lycoris* used in this study have been deposited in the National Genomics Data Center (NGDC), with the BioProject accession numbers PRJCA061346 (genome) and PRJCA061356 (transcriptome), respectively.

## Supplementary Materials

The following supporting information can be downloaded at: https://www.mdpi.com/article/10.3390/plants15081223/s1, Figure S1: Accumulation pattern of kaempferol glycosides in *L. radiata* var. *pumila*. Table S1: Differences in flavonoid metabolites between W and Bp; Table S2: Data quality control; Table S3: Reference alignment results of RNA-seq data; Table S4: Differential gene expression levels of W, Bp, and R in Profile 5; Table S5: Correlation between flavonoid enzyme gene expression and flower color phenotype; Table S6: Correlation between flavonoid enzyme gene expression and flavonoid metabolite accumulation; Table S7: Selecting correlations greater than 0.9 between differentially expressed genes and transcription factors; Table S8: Screening mRNA and lncRNA sequences; Table S9: QRT-PCR for gene expression analysis.
