# Supplementary material for: The LncRNA401-LrWRKY70 Module Regulates the Blue-Purple Flower Color Formation in Lycoris
Source: Plants (Basel). 2026 Apr 16;15(8):1223. doi: 10.3390/plants15081223 (PMC13119879; doi:10.3390/plants15081223)
Supplement: Supplementary file 1 [file plants-15-01223-s001.zip › Supplementary Figure.pdf]

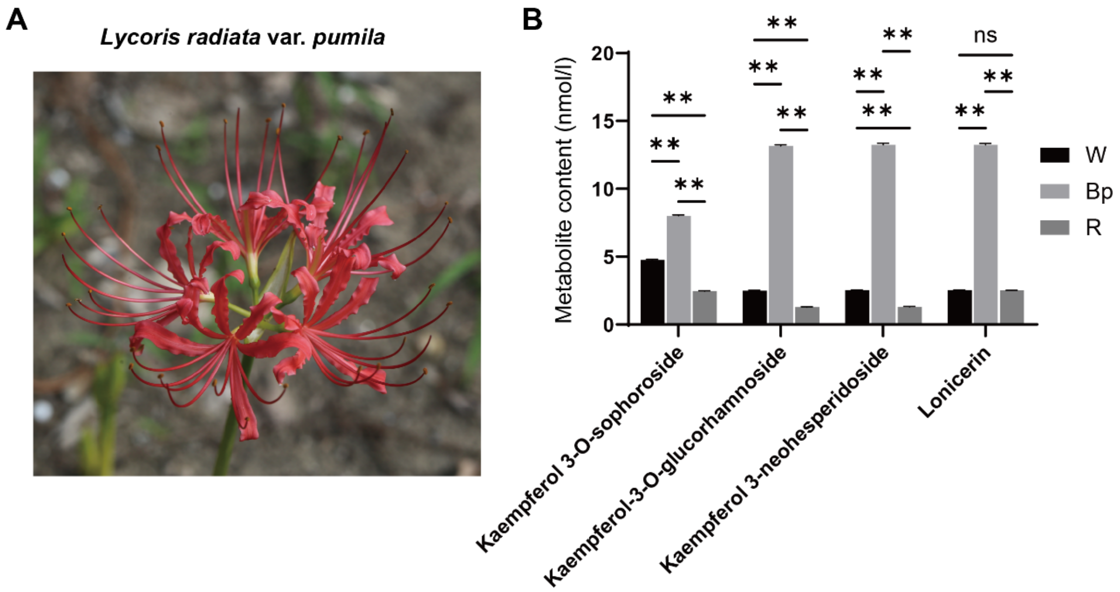


Supplementary Figure S1. Accumulation pattern of kaempferol glycosides in *L. radiata* var. *pumila*. (A) Representative image of *L. radiata* var. *pumila* flower. (B) Quantitative analysis of three kaempferol derivatives and lonicerin in different floral tissues. The results showed that the accumulation levels in the perianth segments (Bp) of *L. sprengeri* were significantly higher than those in the perianth tube (W) of *L. sprengeri* and *L. radiata* var. *pumila* (R). Data are presented as mean ± standard deviation (n=3). $p < 0.01$ compared with the Bp group; ns, no significant difference.
